# Psychometric Properties of the Japanese Version of the STarT Back Tool in Patients with Low Back Pain

**DOI:** 10.1371/journal.pone.0152019

**Published:** 2016-03-22

**Authors:** Ko Matsudaira, Hiroyuki Oka, Norimasa Kikuchi, Yuri Haga, Takayuki Sawada, Sakae Tanaka

**Affiliations:** 1 Department of Medical Research and Management for Musculoskeletal Pain, 22nd Century Medical and Research Center, Faculty of Medicine, The University of Tokyo, Bunkyo-ku, Tokyo, Japan; 2 Clinical Study Support, Inc., Nagoya, Aichi, Japan; 3 Department of Public Health, Aichi Medical University School of Medicine, Nagakute, Aichi, Japan; 4 Department of Orthopaedic Surgery, The University of Tokyo, Bunkyo-ku, Tokyo, Japan; The University of Tokyo Hospital, JAPAN

## Abstract

**Background and Objective:**

The STarT Back Tool uses prognostic indicators to classify patients with low back pain into three risk groups to guide early secondary prevention in primary care. The present study aimed to evaluate the psychometric properties of the Japanese version of the tool (STarT-J).

**Methods:**

An online survey was conducted among Japanese patients with low back pain aged 20–64 years. Reliability was assessed by examining the internal consistency of the overall and psychosocial subscales using Cronbach’s alpha coefficients. Spearman’s correlation coefficients were used to evaluate the concurrent validity between the STarT-J total score/psychosocial subscore and standard reference questionnaires. Discriminant validity was evaluated by calculating the area under the curves (AUCs) for the total and psychosocial subscale scores against standard reference cases. Known-groups validity was assessed by examining the relationship between low back pain-related disability and STarT-J scores.

**Results:**

The analysis included data for 2000 Japanese patients with low back pain; the mean (standard deviation [SD]) age was 47.7 (9.3) years, and 54.1% were male. The mean (SD) STarT-J score was 2.2 (2.1). The Cronbach’s alpha coefficient was 0.75 for the overall scale and 0.66 for the psychosocial subscale. Spearman’s correlation coefficients ranged from 0.30 to 0.59, demonstrating moderate to strong concurrent validity. The AUCs for the total score ranged from 0.65 to 0.83, mostly demonstrating acceptable discriminative ability. For known-groups validity, participants with more somatic symptoms had higher total scores. Those in higher STarT-J risk groups had experienced more low back pain-related absences.

**Conclusions:**

The overall STarT-J scale was internally consistent and had acceptable concurrent, discriminant, and known-groups validity. The STarT-J can be used with Japanese patients with low back pain.

## Introduction

Low back pain (LBP) is a major musculoskeletal problem in the general population from childhood to older adulthood, affecting more than 632 million people worldwide [1]. The 2010 Global Burden of Diseases, Injuries, and Risk Factors Study reported that LBP was the leading cause of disability among 291 diseases and injuries globally, and LBP ranked as the highest global cause of years lived with disability [2]. This highlights the high prevalence of LBP worldwide, and may also reflect the difficulty of successful LBP management. In primary care, approximately 85% of patients with LBP have no specific underlying causes or pathology [3]. Patients with non-specific LBP often experience recurrent pain, and the majority of these patients suffer from chronic pain [4–5]. Recurrent and chronic LBP may result in a serious social and economic burden.

Psychological factors have been widely acknowledged as contributors to the chronicity of LBP [6–7]. These factors include pain catastrophizing, fear-avoidance beliefs, and psychological distress. A number of previous reports suggested an association between psychological factors and poor long-term outcomes [5, 8–9]. In primary care, cognitive behavioral therapy focused on psychological factors is a dominant treatment approach for people with LBP [5]. To provide efficient, targeted care, it is becoming common to stratify patients with LBP according to their risk for poor long-term outcomes [10]. Significant clinical benefits and cost-effectiveness of stratified care compared with non-stratified physiotherapy practice have been demonstrated in a randomized clinical trial [11].

**Fig 1:**
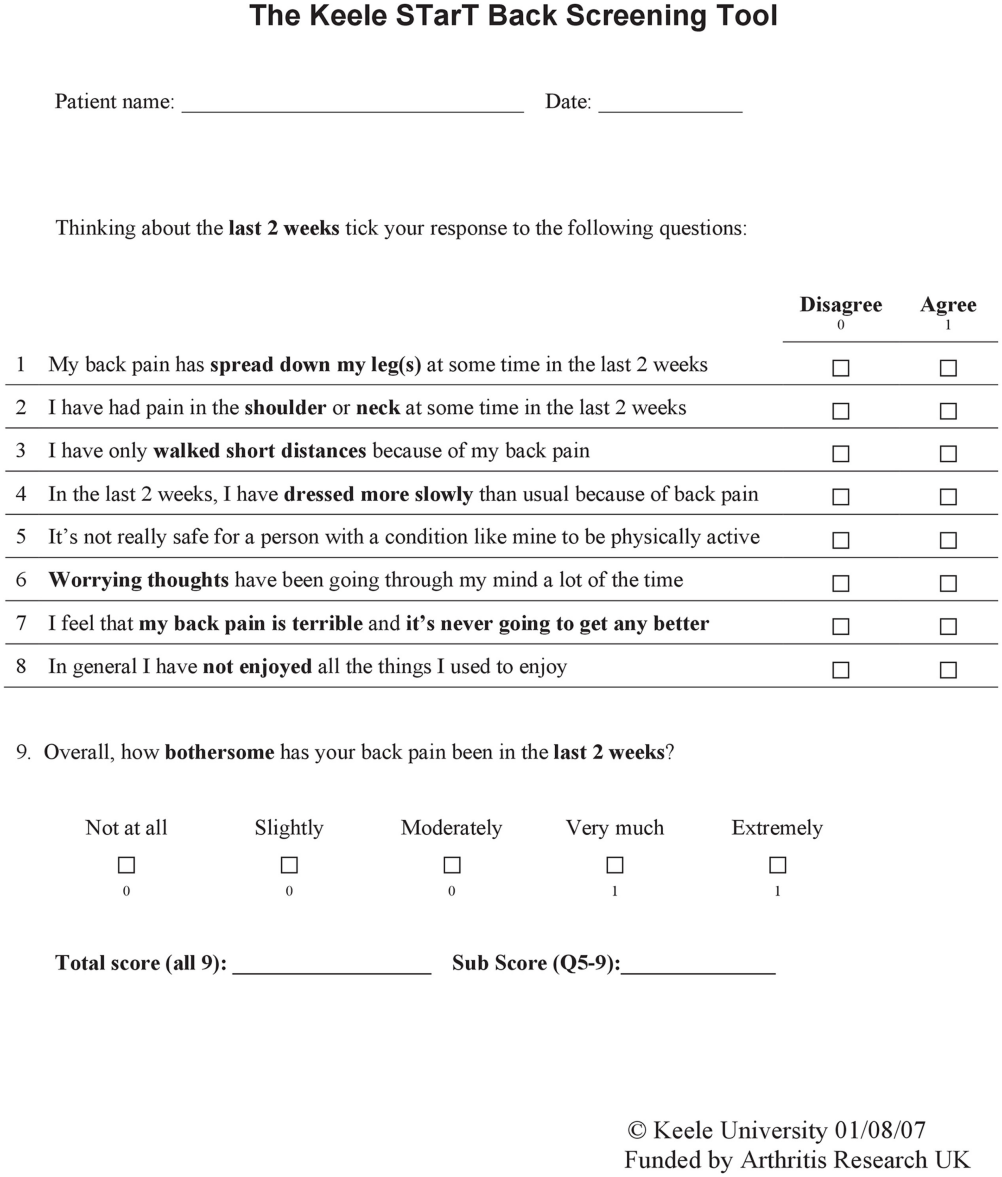
STarT Back Tool. Response options for items 1–8 are “disagree” (0 points) or “agree” (1 point). Responses to item 9 are on a scale of 1–5: “not at all,” “slightly,” “moderately,” “very much,” or “extremely.” The first three options (“not at all,” “slightly,” and “moderately”) are scored as 0, and the remaining two options (“very much” and “extremely”) are scored as 1. Items 1–4 constitute the physical subscale. Items 5–9 constitute the psychosocial subscale.

**Fig 2:**
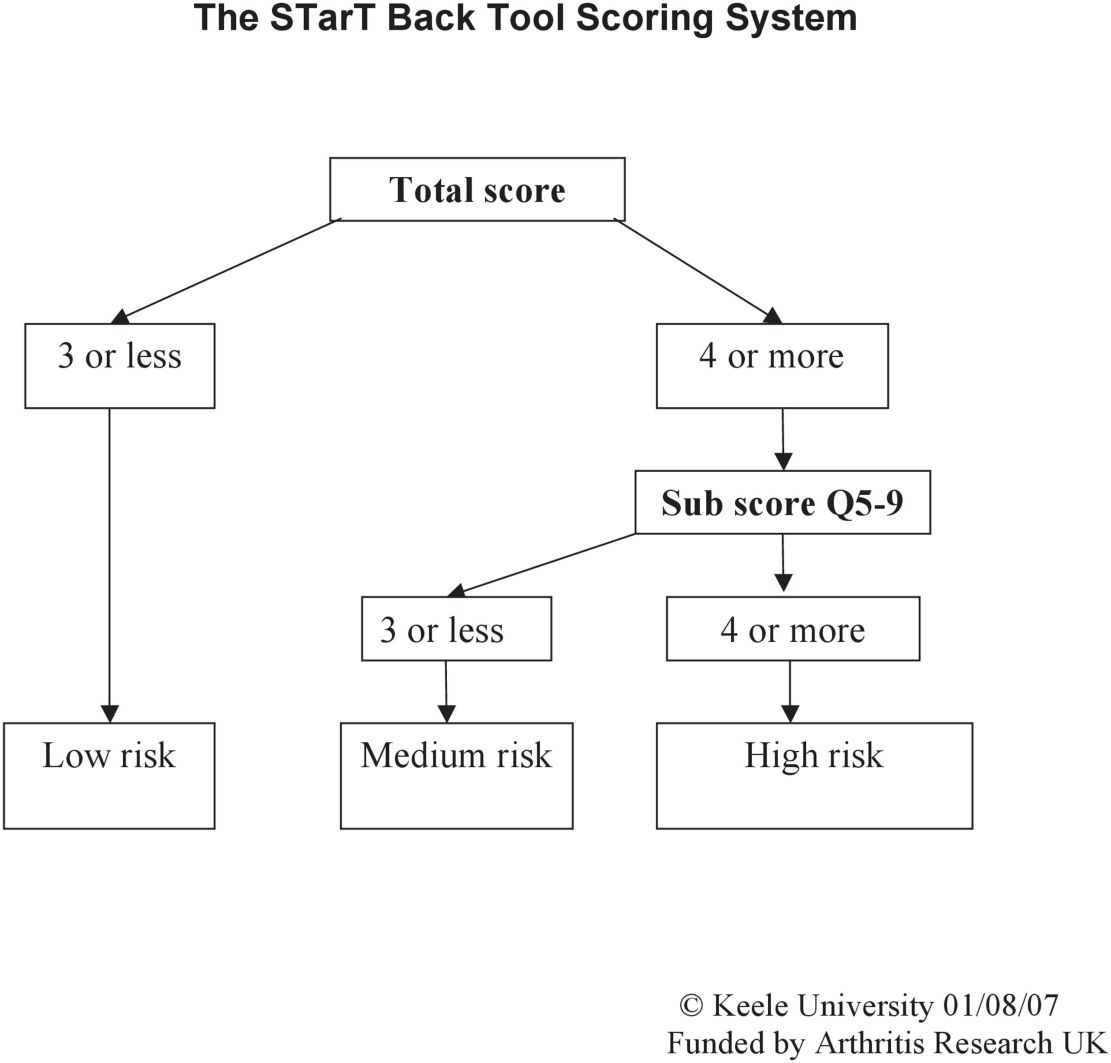
STarT Back Tool risk stratification. Sub score Q5–9: psychosocial subscale.

The STarT Back Tool (STarT) has been widely used to stratify patients with LBP according to risk for chronicity (Fig 1). The STarT was originally developed as a screening tool for prognostic indicators of back pain to help primary care clinical decision-making in the UK [12]. The STarT consists of 9 items. Items 1–4 evaluate physical factors, and items 5–9 assess psychosocial factors. The STarT classifies patients into three risk groups: patients with a total score of 0–3 are classified as low-risk; patients with a total score of ≥ 4 but a psychosocial subscore of ≤ 3 as medium-risk; and patients with a psychosocial subscore of ≥ 4 are classified as high-risk [12] (Fig 2). Targeted treatments have been developed for patients in each risk group: a minimal intervention by general practitioners or physiotherapists for the low-risk group, physiotherapy to address pain and disability for the medium-risk group, and psychologically-informed physiotherapy to address pain and disability as well as psychosocial obstacles to recovery for the high-risk group [11, 13, 14].

Although the STarT has been translated into various languages, no validated Japanese version was available. In our previous study, we translated the original English version of the STarT into Japanese (STarT-J) and linguistically validated it [15]. As a next step, we conducted online surveys with Japanese people with LBP to evaluate the psychometric properties of the STarT-J. The present analysis aimed to evaluate the reliability and validity of the STarT-J in a large number of Japanese people with LBP, using cross-sectional data from these surveys.

## Materials and Methods

### Study population

To assess the psychometric properties of the STarT-J, we conducted online surveys collecting information on LBP in the Japanese population in January and February, 2014. Participants were recruited from an online panel provided by an Internet research company, UNITED, Inc. (Tokyo, Japan), which included approximately 1.25 million individuals aged 20–64 years registered as research volunteers. From these volunteers, 965,919 individuals were randomly selected and invited by e-mail to complete an online questionnaire on health problems associated with pain (first survey). We obtained 52,842 responses by the end of January 2014. Of these initial respondents, those who had LBP in the last 4 weeks were invited to complete another online questionnaire (secondary survey). LBP was defined as pain in the lower back experienced in the last 4 weeks that lasted for more than 1 day, according to the standard definition of LBP proposed by Dionne et al. [16]. Pain associated with menstruation or pregnancy and pain during a feverish illness were excluded. A diagram showing the lower back area (between the inferior costal margin and gluteal folds) was provided in the questionnaire. The secondary survey closed on 7 February 2014, when the total number of responses reached 2000. The mean (standard deviation [SD]) age of respondents in the secondary survey was 47.7 (9.3) years and 54.1% were male. We conducted two subsequent surveys, 6 and 24 weeks after the secondary survey, to follow up respondents and investigate their LBP condition. In the present analysis, we analyzed secondary survey data to evaluate the psychometric properties of the STarT-J.

We obtained approval from the Medical/Ethics Review Board of the Japan Labour Health and Welfare Organization, Kanto Rosai Hospital (Approval number: 2012–22). Participation was voluntary, and no personal information was collected. Although no written informed consent was obtained, submitting a completed questionnaire was considered as evidence of consent. Potential participants first read an explanation of the aim of the survey and only those who agreed to participate could proceed to the questionnaire. As an incentive, participants received reward points for online shopping from the Internet research company.

### Development of the linguistically-validated STarT-J

In our previous study [15], the STarT was translated into Japanese and linguistically validated in a general cross-cultural adaptation process [17–19]. This process occurred in three steps: (1) forward-translation (English to Japanese), (2) back-translation (Japanese to English), and (3) cognitive debriefing. In the third step, we conducted a pilot study to assess if the questions and response scales were understandable and correctly interpreted by Japanese patients. After considering their feedback, and consultation with a specialist as necessary, we published the STarT-J [15].

### Measures

We included a number of measures in the online questionnaires.

#### Pain

The degree of pain associated with LBP during the last 4 weeks was assessed by a numerical rating scale (NRS), ranging from 0 (no pain at all) to 10 (the worst pain imaginable).

#### Disability caused by LBP

We used the Roland—Morris Disability Questionnaire (RDQ) to assess the LBP-related disability participants experienced in their daily lives. The RDQ comprises 24 Yes/No questions. The total score ranges from 0 to 24, with a higher score indicating greater disability. In this study, we used the Japanese version of the RDQ, for which the reliability and validity have been previously confirmed [20].

#### Fear-avoidance beliefs

Fear of pain can lead to avoidance of physical activity, an important indicator of a poor long-term LBP prognosis. The Fear-Avoidance Belief Questionnaire (FABQ), consisting of physical activity and work subscales, is widely used to assess fear-avoidance beliefs [21]. We used the FABQ physical activity subscale (FABQ-PA). The FABQ-PA score ranges from 0 to 30; a higher score indicates a stronger fear-avoidance belief. We also used the Tampa Scale of Kinesiophobia (TSK) [22–23], originally developed to measure the fear of movement or injury. The total TSK score sums the scores of 17 items (each rated on a scale of 1–4), and ranges from 17 to 68. A higher score indicates a higher level of kinesiophobia.

#### Catastrophizing

Pain catastrophizing is also an important indicator of poor LBP prognosis. Catastrophizing was assessed using the Pain Catastrophizing Scale (PCS), originally developed to measure negative attitudes toward pain involving rumination, helplessness, and magnification. The PCS consists of 13 items. The total score ranges from 0 (no catastrophizing) to 52 (greater catastrophizing). We used the Japanese version of the PCS, for which the reliability and validity have been previously confirmed [24].

#### Depression and anxiety

A 14-item self-assessment scale, the Hospital Anxiety and Depression Scale (HADS), was used to measure anxiety and depression. The HADS comprises anxiety and depression subscales, each with seven items. The total score ranges from 0 to 21, with a higher score indicating more mental distress. The validity and reliability of the Japanese version of the HADS have been previously confirmed [25].

#### General health status

The EuroQol 5 Dimension (EQ-5D) [26] is an instrument that provides a simple, descriptive profile and single index value for general health status. The index score is derived from conversion of all responses, and ranges from −0.11 to 1.00. A score of 1 means “perfect health” and a score of 0 denotes “death.”

#### Somatic symptoms

Somatization was assessed using the 7-item somatization subscale from the Brief Symptom Inventory (BSI) [27]. Seven symptoms (faintness or dizziness, pains in the heart or chest, nausea or upset stomach, trouble getting your breath, numbness or tingling in parts of the body, feeling weak in parts of the body, hot or cold spells) are rated on a 5-point scale: “not at all,” “a little bit,” “moderately,” “quite a bit,” and “extremely.” We used the linguistically validated Japanese version of the BSI-somatization subscale [28].

### Data analyses

Participants’ demographic and clinical characteristics were summarized using descriptive statistics. To examine floor and ceiling effects, percentages of respondents with total scores of 0 and 9 were calculated. Floor and ceiling effects were considered to exist when more than 15% of respondents had the lowest or highest possible score [29]. To examine the reliability of the STarT-J, we evaluated internal consistency by calculating Cronbach’s alpha coefficients for the overall scale and the psychosocial subscale. An alpha index more than 0.70 is considered to indicate satisfactory internal consistency [30].

Concurrent validity was evaluated by measuring correlations between the previously described reference instruments and the STarT-J total score and psychosocial subscore using Spearman’s correlation coefficients. Correlation coefficients were evaluated according to the criteria for correlation strength in psychometric validation proposed by Cohen: 0.10 representing a weak, 0.30 a moderate, and 0.50 a strong correlation [31].

To assess discriminant validity, we calculated the area under the curves (AUCs) for the total scores and psychosocial subscores against the reference standards. We defined cases using the following cut-off values: a RDQ score of ≥ 7 for disability, a PCS score of ≥ 20 for catastrophizing, a TSK score of ≥ 41 for fear-avoidance beliefs, and a HADS score of ≥ 8 for depression and anxiety. In addition, a single question was used to determine the presence of referred leg pain within the last 4 weeks. Discriminative ability was interpreted according to the same criteria as used in the original STarT study: 0.70 to < 0.80 indicating acceptable discrimination, 0.80 to < 0.90 indicating excellent discrimination, and ≥ 0.90 indicating outstanding discrimination [12].

For known-groups validity, to test whether the STarT-J scores differentiated participants with known differences, we examined 1) total scores among the groups with a different number of somatic symptoms, and 2) the number of absences due to LBP among the three risk groups (low, medium, and high) using the Jonckheere—Terpstra test. If participants responded “moderately,” “quite a bit,” or “extremely” to a BSI item, they were considered to have that somatic symptom. Participants were then categorized into three groups according to the number of somatic symptoms: no symptoms, one symptom, and two or more symptoms. With respect to the number of absences, days on which participants could not perform housework were counted, as well as absences from work. It was hypothesized that participants with more somatic symptoms would have higher total scores, and that participants in the high-risk group would have experienced more LBP-related absences.

All statistical analyses were performed using SAS version 9.3 (SAS Institute, Cary, NC, USA). The level of significance was set at 0.05.

## Results

### Participant characteristics

The present analysis included data for 2000 Japanese patients with LBP. Table 1 presents a summary of participants’ demographic and clinical characteristics. The mean (SD) age was 47.7 (9.3) years; 54.1% of participants were male. More than half (53.7%) of the participants had experienced LBP for more than 1 year. Most participants (92%) experienced recurrent LBP, and more than half (52.9%) reported having LBP 10 times or more.

**Table 1:** Participant characteristics: psychometric testing of the STarT-J (n = 2000).

Characteristics	n (%)	Mean (SD)
Sex		
Male	1081 (54.1)	
Female	919 (46.0)	
Age (years)		47.7 (9.3)
BMI ≥ 25 (kg/m^2^)	506 (25.3)	
Duration of low back pain		
< 2 weeks	350 (17.5)	
≥ 2 weeks, < 1 month	188 (9.4)	
≥ 1, < 3 months	184 (9.2)	
≥ 3, < 6 months	90 (4.5)	
≥ 6 months, < 1 year	115 (5.8)	
≥ 1, < 3 years	200 (10.0)	
≥ 3 years	873 (43.7)	
Number of recurrence		
1	160 (8.0)	
2	135 (6.8)	
3–4	340 (17.0)	
5–9	308 (15.4)	
≥10	1057 (52.9)	
STarT-J score		2.2 (2.1)
RDQ score		4.2 (4.7)
FABQ-PA score		12.9 (4.7)
TSK score		41.0 (6.5)
PCS total score		21.6 (10.0)
PCS rumination		10.6 (4.3)
PCS helplessness		6.2 (4.2)
PCS magnification		4.7 (2.7)
HADS total score		17.2 (6.7)
HADS anxiety		8.7 (3.4)
HADS depression		8.5 (4.1)
EQ-5D index score		0.78 (0.16)
NRS for low back pain		4.2 (1.8)

Values are n (%), or mean (SD).

STarT-J, the Japanese version of the STarT Back Tool; BMI, body mass index; RDQ, Roland—Morris Disability Questionnaire; FABQ-PA, Fear-Avoidance Belief Questionnaire Physical Activity Subscale; TSK, Tampa Scale for Kinesiophobia; PCS, Pain Catastrophizing Scale; HADS, Hospital Anxiety and Depression Scale; EQ-5D, EuroQol 5 Dimension; NRS, numerical rating scale.

### Scores of the measures

The mean (SD) score for the STarT-J was 2.2 (2.1). No remarkable ceiling effect was observed as 0.9% of participants had the highest score of 9. However, a floor effect was observed as 23.4% of participants had the lowest score of 0. The score distribution for each item is shown in Table 2. Participants were classified into three risk groups according to their STarT-J score: 1557 (77.9%) into the low-risk group, 294 (14.7%) into the medium-risk group, and 149 (7.5%) into the high-risk group.

**Table 2:** Score distribution of STarT-J items and risk group distribution (n = 2000).

Item	Number of participants who answered “agree” (1 point)
	n (%)
1	442 (22.1)
2	1069 (53.5)
3	317 (15.9)
4	264 (13.2)
5	574 (28.7)
6	652 (32.6)
7	425 (21.3)
8	351 (17.6)
9	239 (12.0)
Risk group distribution	
Low-risk	1557 (77.9)
Medium-risk	294 (14.7)
High-risk	149 (7.5)

Values are n (%).

STarT-J: The Japanese version of the STarT Back Tool. For item 9, answers of “very much” and “extremely” were scored as 1 point, and were counted as “agree”; the answers “not at all,” “slightly,” and “moderately” were scored as 0 points, and were not included.

### Reliability

The Cronbach’s alpha coefficients were 0.75 for the overall scale and 0.66 for the psychosocial subscale.

### Concurrent validity

To examine concurrent validity, Spearman’s correlation coefficients were used to measure correlations between the STarT-J total score/psychosocial subscore and the pain NRS, RDQ, FABQ-PA, TSK, PCS, HADS, and the EQ-5D (Table 3). The correlation coefficients for the total score ranged from 0.30 (HADS depression) to 0.59 (RDQ), demonstrating a moderate to strong correlation with these reference standards. Similarly, correlation coefficients for the psychosocial subscore ranged from 0.33 (FABQ-PA) to 0.54 (RDQ), demonstrating a moderate to strong correlation. Both the total score and psychosocial subscore were strongly negatively correlated with the EQ-5D (γ = −0.56 and γ = −0.53, p < 0.0001). In terms of the correlation with psychosocial measures, the total score was moderately correlated with the TSK (γ = 0.49), whereas the psychosocial subscore was strongly correlated (γ = 0.53). Moderate correlation coefficients were observed for both the total score and psychosocial subscore with the PCS (γ = 0.46 and γ = 0.49) and the HADS (γ = 0.40 and γ = 0.45) (p < 0.0001 for all).

**Table 3:** Spearman’s correlation coefficients for the STarT-J and related measures.

Measures	Total score	Psychosocial subscore
	Coefficients (95% CI)	Coefficients (95% CI)
RDQ	0.59 (0.56–0.62)	0.54 (0.51–0.57)
FABQ-PA	0.34 (0.30–0.37)	0.33 (0.29–0.37)
TSK	0.49 (0.45–0.52)	0.53 (0.50–0.56)
PCS total	0.46 (0.42–0.49)	0.49 (0.46–0.52)
PCS rumination	0.43 (0.40–0.47)	0.44 (0.41–0.48)
PCS helplessness	0.39 (0.35–0.43)	0.43 (0.39–0.46)
PCS magnification	0.40 (0.36–0.44)	0.43 (0.39–0.47)
HADS total	0.40 (0.36–0.44)	0.45 (0.41–0.48)
HADS anxiety	0.42 (0.38–0.46)	0.46 (0.42–0.49)
HADS depression	0.30 (0.26–0.34)	0.35 (0.31–0.39)
EQ-5D	−0.56 (−0.59 –−0.52)	−0.53 (−0.56 –−0.50)
NRS for low back pain	0.42 (0.38–0.46)	0.39 (0.35–0.42)

Note: p < 0.0001 for all correlation coefficients. STarT-J, the Japanese version of the STarT Back Tool; CI, confidence interval; RDQ, Roland—Morris Disability Questionnaire; FABQ-PA, Fear-Avoidance Belief Questionnaire Physical Activity Subscale; TSK, Tampa Scale for Kinesiophobia; PCS, Pain Catastrophizing Scale; HADS, Hospital Anxiety and Depression Scale; EQ-5D, EuroQol 5 Dimension; NRS, numerical rating scale.

### Discriminant validity

To assess discriminant validity, AUCs were calculated for the total score and psychosocial subscore against the cases defined by the reference standards (Table 4). The AUCs for the total score were all above 0.70, indicating acceptable to excellent discriminative ability, with the exception of depression and anxiety (0.65). For the psychosocial subscore, the AUCs ranged from 0.67 (depression and anxiety) to 0.79 (disability), indicating poor to acceptable discriminative ability.

**Table 4:** AUCs for STarT-J total score and psychosocial subscore against reference standards.

Reference standards	Case definition	Total score	Psychosocial subscore
		AUC (95% CI)	AUC (95% CI)
Disability	RDQ score ≥ 7	0.83 (0.81–0.85)	0.79 (0.77–0.82)
Referred leg pain	Yes	0.76 (0.73–0.79)	0.68 (0.65–0.72)
Fear-avoidance belief	PCS score ≥ 20	0.71 (0.69–0.73)	0.72 (0.70–0.74)
Catastrophizing	TSK score ≥ 41	0.74 (0.72–0.76)	0.75 (0.73–0.77)
Depression and anxiety	HADS score ≥ 8	0.65 (0.63–0.68)	0.67 (0.65–0.69)

AUC, area under the curve; STarT-J, the Japanese version of the STarT Back Tool; CI, confidence interval; RDQ, Roland—Morris Disability Questionnaire; PCS, Pain Catastrophizing Scale; TSK, Tampa Scale for Kinesiophobia; HADS, Hospital Anxiety and Depression Scale.

### Known-groups validity

**Fig 3:**
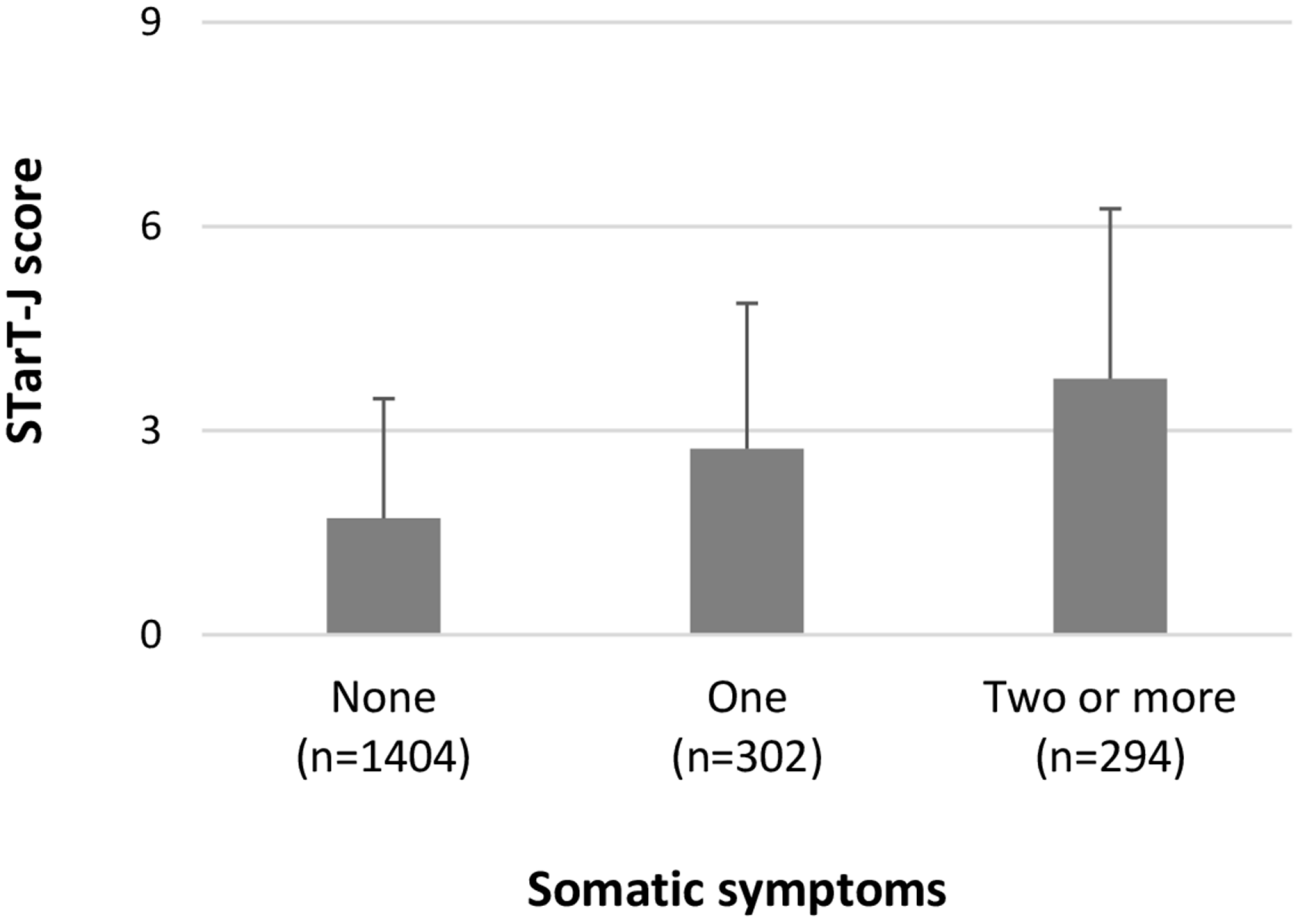
Mean STarT-J scores for participants with different numbers of somatic symptoms. The linear trend was tested using the Jonckheere-Terpstra test (p < 0.0001). STarT-J: The Japanese version of the STarT Back Tool. Number of somatic symptoms was assessed by the Brief Symptom Inventory somatization scale: a response of “moderately,” “quite a bit,” or “extremely” to an item was interpreted as the presence of that somatic symptom, and thus counted.

**Fig 4:**
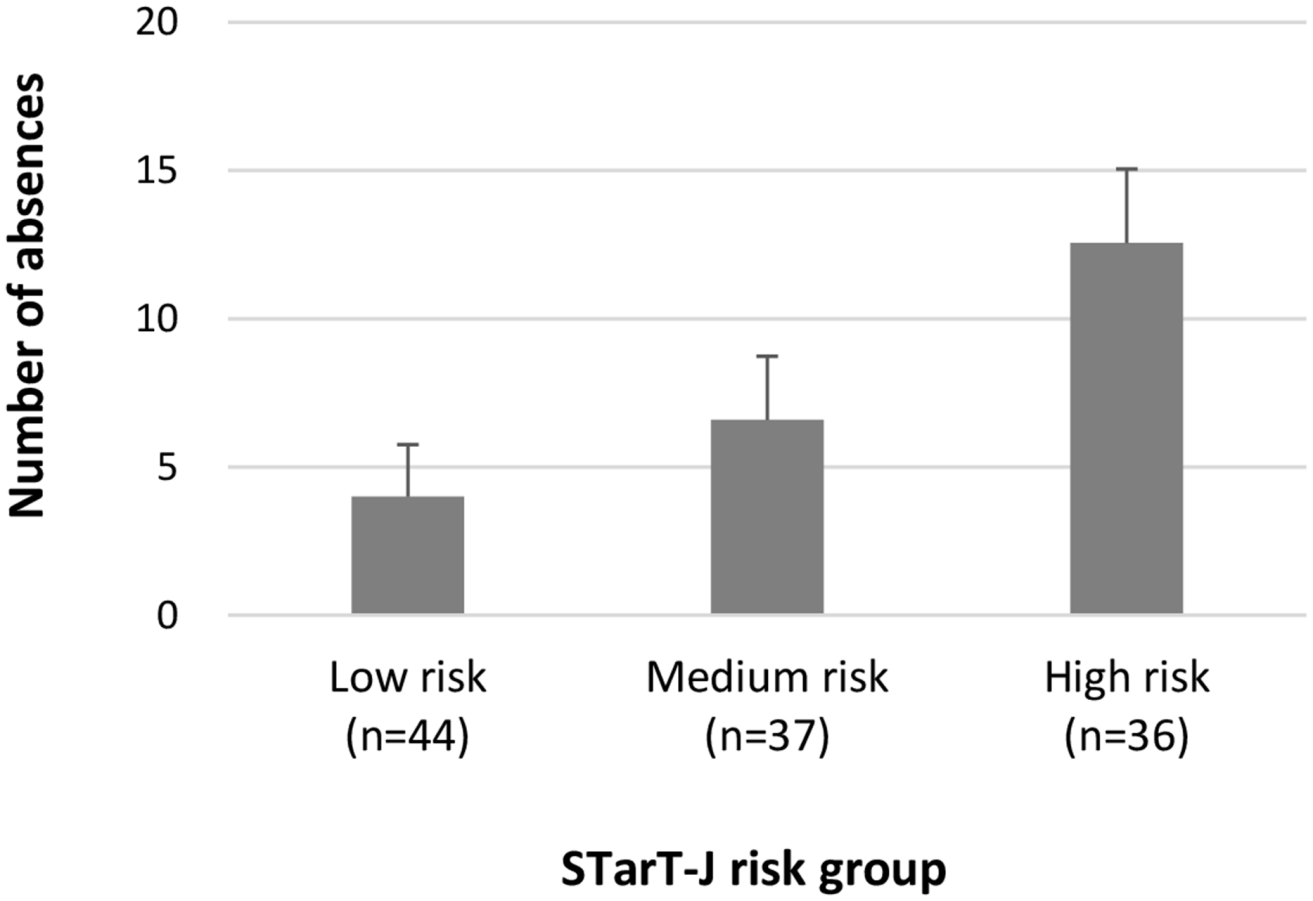
Mean number of absences for the three STarT-J risk groups. The linear trend was tested using the Jonckheere-Terpstra test (p < 0.0001). STarT-J: The Japanese version of the STarT Back Tool.

We examined the STarT-J total scores and risk groups among participants with known-differences. As hypothesized, participants with more somatic symptoms had higher total scores. The mean (SD) score of participants with no somatic symptoms was 1.71 (1.76), one somatic symptom was 2.73 (2.14), and two or more somatic symptoms was 3.76 (2.50) (Fig 3). A linear increasing trend in total score across groups with an increasing number of somatic symptoms was observed (Jonckheere-Terpstra test, p < 0.0001). With respect to the associations between risk groups and the number of absences, participants in the high-risk group reported a larger number of absences (Fig 4). The mean (SD) LBP-related absences in the low-risk group was 4.0 (5.4) days, 6.6 (8.3) days in the medium-risk group, and 12.6 (11.1) days in the high-risk group. A linear increasing trend in the number of absences across the risk groups was observed (Jonckheere-Terpstra test, p < 0.0001).

## Discussion

In this analysis, we evaluated the psychometric properties of the STarT-J. In summary, the overall scale of the STarT-J was internally consistent, and the STarT-J had acceptable concurrent validity, discriminant validity, and known-groups validity in Japanese patients with LBP.

The Cronbach’s alpha coefficient for the overall scale (0.75) demonstrated sufficient internal consistency, and was similar to the original and other language versions: 0.79 for the original [12], 0.74 for the French [32], 0.74 for the Brazilian Portuguese [33], 0.82 for the Iranian [34], and 0.83 for the Persian [35] versions. Although these results could not be compared directly because the study methods varied, the similar values support that the overall scale of the STarT-J is internally consistent and no items are redundant. The Cronbach’s alpha coefficient for the psychosocial subscale was 0.66, below the value of 0.70 considered necessary to claim the subscale is internally consistent. However, it should be taken into consideration that the coefficient for the subscale was also lower than for the overall scale in the original version, although it was still 0.74 [12].

To assess concurrent validity, we analyzed the correlations between the STarT-J and reference standards (the pain NRS, RDQ, FABQ-PA, TSK, PCS, HADS, and EQ-5D). Overall, the Spearman’s correlation coefficients indicated that both the total score and the psychosocial subscore were moderately to strongly correlated with these existing scales. In particular, the STarT-J total score was strongly correlated with the RDQ (γ = 0.59). Similar results were observed in the German (γ = 0.55) [36], French (γ = 0.74) [32], and Persian (γ = 0.811) [35] versions. Although a direct comparison cannot easily be made, these similar results reinforce the concurrent validity of the STarT-J.

Discriminant validity was assessed by calculating the AUCs for the total score and the psychosocial subscore. For the total score, the AUCs for disability and referred leg pain were both higher than the AUCs for fear-avoidance beliefs, catastrophizing, and depression and anxiety. This demonstrated that the total score better discriminated cases defined by physical reference standards. However, for the psychosocial subscore, the AUCs for fear-avoidance beliefs, catastrophizing, and depression and anxiety were not remarkably higher than AUCs for the physical reference cases. These AUCs for the psychosocial reference cases were similar to those for the total score, indicating the psychosocial subscale might discriminate cases defined by the psychosocial reference standards at a similar level to the overall scale. A similar trend was observed in the original STarT [12], although overall, the AUCs were higher compared with the STarT-J.

To assess known-groups validity, we investigated relationships between total scores and the number of somatic symptoms, and between risk groups and the number of absences. Participants with more somatic symptoms had higher total scores, and those in the high-risk group had experienced greater LBP-related disability. This demonstrated that the STarT-J can differentiate patients with different levels of LBP-related problems.

The present study has some limitations. First, we did not examine the test—retest reliability. The intra-class, test—retest reliability over specific time intervals should therefore be evaluated in a future study. Second, the analysis might have included patients not targeted by the STarT, that is, patients who had specific causes of LBP. The diagnostic triage for LBP is to classify LBP into one of three categories: LBP with specific pathologic change (“red flag”), LBP with sciatica/radicular syndrome, or non-specific LBP [37]. According to this classification, six of the participants in the present analysis were probable “red flags,” 308 had radicular syndrome, and the remaining 1686 participants were considered to have non-specific LBP. As the original study included patients with non-specific LBP who had referred leg pain [12], the STarT is considered applicable to patients with LBP potentially associated with sciatica/radicular syndrome. Therefore, assuming diagnoses were accurate, most participants probably fit into the STarT target group. However, it should be noted that these diagnoses might not necessarily be accurate as they were based on participants’ self-report. Third, the study population might not be consistent with the primary care population. Our study included more low-risk participants and less high-risk participants compared with the original study [12]. This might be because we recruited from a general Japanese population registered with an online panel rather than from patients in hospitals. Our study population would therefore represent the general Japanese population with LBP. As the observed floor effect suggests, more patients might have LBP that was not sufficiently severe to require hospital care. Although our study population was broader than the primary care population, the percentage of patients with non-specific LBP was similar to that observed in primary care settings. In our study, 1686 participants (84.3%) probably had non-specific LBP. In primary care, approximately 85% of patients with LBP have non-specific LBP [3]. Therefore, our study population resembled the primary care population in terms of the distribution of non-specific LBP. Fourth, as this was a cross-sectional study, it did not assess the ability of the STarT-J to predict chronicity of LBP. To assess its predictive ability, longitudinal studies will be necessary to investigate associations between risk groups and long-term outcomes of patients with LBP.

In the present analysis, we evaluated the psychometric properties of the STarT-J to enable Japanese clinicians to use the scale in the early stages of LBP. The STarT is a simple and quick tool, and is suitable for use in primary care settings. Stratified care is a dominant approach in the management of LBP [10]. Stratified care based on the STarT risk groups has been shown to be clinically and economically beneficial for patients with LBP [11, 38]. Therefore, we expect that the STarT-J may facilitate early stratified care in primary care settings in Japan. This may alleviate the physical, social, and economical burden of LBP in the Japanese population.

In conclusion, acceptable internal consistency for the overall STarT-J scale demonstrated the reliability of the STarT-J in Japanese patients with LBP; acceptable concurrent validity, discriminant validity, and known-groups validity demonstrated the validity. In a subsequent analysis, the ability of the STarT-J to predict chronicity of LBP will be examined using longitudinal data, to validate its clinical use in Japanese patients.
